# Improvement in Accuracy and Concordance of American Society of Anesthesiologist's Physical Status (ASA-PS) Scoring Assignment over a 11 Year Time Period Using Patient BMI as a Comorbidity Finding

**DOI:** 10.1155/2024/6989174

**Published:** 2024-05-22

**Authors:** Matthew W. Dyer, Benjamin T. Kor, Nathan T. Kor, Andrew C. Hanson, Jennifer J. Kor, Todd M. Kor, Thomas M. Stewart, Hans P. Sviggum

**Affiliations:** ^1^Department of Anesthesiology and Perioperative Medicine, Mayo Clinic, Rochester, MN, USA; ^2^Department of Clinical Trials and Biostatistics, Mayo Clinic, Rochester, MN, USA; ^3^Kentucky College of Osteopathic Medicine, University of Pikeville, Pikeville, KY, USA

## Abstract

**Background:**

Anesthesia providers categorize patients utilizing the American Society of Anesthesiologists Physical Status (ASA-PS) classification originally created by the ASA in 1941. There is published variability and discordance among providers when assigning patient ASA scores in part due to the subjectivity of scoring utilizing patient medical conditions, but variability is also found using objective findings like BMI. To date, there are few studies evaluating the accuracy of anesthesia providers' ASA assignment based on objective body mass index (BMI) alone. The aim of this retrospective chart review is to determine improvement in accuracy of anesthesia providers to correctly assign patient ASA scores, based on BMI criteria added to the ASA-PS in October of 2014, utilizing a multifaceted strategy including creation of an active finance committee in the fall of 2015, multiple e-mail communications about the updated definitions and recommendations for ASA-PS scoring in the fall of 2015 and spring of 2016, a department grand rounds presentation in February 2016, placement of laminated copies of the ASA definitions and recommendations in the anesthesia chartrooms, and the development of a tool embedded into our EMR providing a recommendation of ASA-PS based on patient comorbidity findings.

**Methods:**

After attaining IRB approval, all eligible patients over the age of 18 who had surgical procedures under general anesthesia at Mayo Clinic in Rochester, MN, between January 1, 2010, and December 31, 2020, were retrospectively analyzed. A segmented logistic regression model was used to estimate the trends (per-year change in odds) of ASA under classification according to severity of obesity during 3 epochs: preimplementation (2010–2014), implementation (2015), and postimplementation (2016–2020).

**Results:**

A total of 16,467 patients of the 200,423 (8.2%) patients with obesity (class 1, 2, and 3) were underscored based on BMI alone. Accuracy of ASA-PS classification, as it pertains to BMI alone, was found to show meaningful improvement year-to-year following the updated ASA-PS guidelines with examples released in October of 2014 (*P* < 0.001). Most of the improvement occurred in 2015–2017 with relatively little between-year variability in the rate of underscoring from 2017–2020.

**Conclusion:**

Despite updated ASA-PS published guidelines, providers may still be unaware of the updated guidelines and inclusion of examples used within the ASA-PS classification system. Accuracy of scoring did improve annually following the release of the updated guidelines with examples as well as department-wide educational activities on the topic. Additional education and awareness should be offered to those responsible for preanesthesia evaluation and assignment of ASA-PS in patients to improve accuracy as it pertains to BMI.

## 1. Introduction

The American Society of Anesthesiologists (ASA) Physical Status (PS) classification (ASA-PS) was first described by Saklad [1]. The original intent of the classification system was for statistical research of patients undergoing anesthesia. Over time, there have been multiple revisions to the ASA-PS [2–5], and research conducted related to the accuracy and concordance of this scoring system [6–23]. The current ASA-PS describes 6 different categories related to the patient's current physical state and medical comorbidities [5]. This scoring tool has been used not only as a physical status scoring system for preoperative evaluation but also in policy-making, performance evaluation, allocation of resources, quality metrics, and reimbursement of anesthesia services in many commercially insured patient populations [24].

Studies looking at inter-rater reliability and concordance of ASA-PS have consistently shown wide variation in scoring between anesthesia and nonanesthesia providers alike. For example, Owens et al. found significant discordance in scoring using the ASA-PS when 304 randomly picked anesthesiologists were sent surveys asking them to classify patients using the ASA-PS definitions, as less than 60% of patients were scored identically [6]. One of the most perplexing causes of potential scoring discordance is body habitus. Obesity has long been understood as a complicating factor for surgical outcomes and the management of anesthesia [3, 16, 18]. However, there is a paucity of research to date to evaluate the accuracy of ASA-PS in scoring patients based on obesity.

On October 15, 2014, and again on December 13, 2020, the ASA Committee on Economics released updated ASA-PS guidelines that included examples of various physical states and comorbidities which now include adult, pediatric, and obstetric examples [5]. The new guidelines listed several examples of conditions to help reduce subjectivity and provider-provider discordance in scoring, including examples of obesity. Class 1 and 2 obesity are grouped together in an ASA-PS category using body mass index (BMI) defined as BMI 30.0–39.9 kg/m^2^ and carry a minimum ASA-PS of II. Class 3 obesity is defined as BMI >40.0 kg/m^2^ and carries a minimum ASA-PS of III. Utilizing these examples, Fielding-Sing et al. found that 4.8% of patients with class 1 or 2 obesity and 27.6% of patients with class 3 obesity were underclassified by ASA-PS [21]. Their study suggested that the published examples may have had a limited impact on improving accuracy and concordance.

**Figure 1:**
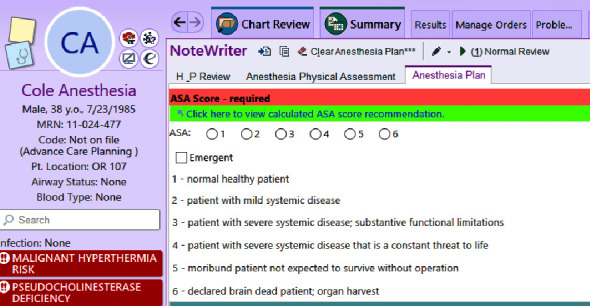
Image from our EMR preanesthesia note creator on a sample patient showing a hyperlink to the ASA scoring tool clinicians may utilize to assist with ASA-PS scoring.

**Figure 2:**
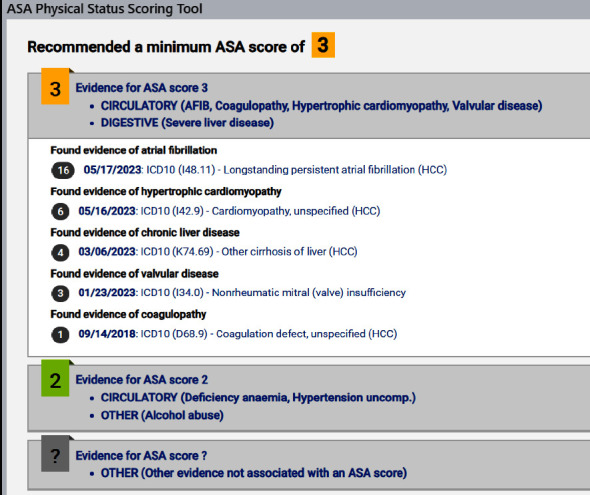
Comorbidities identified by the scoring tool from a sample patient and a recommended ASA-PS score generated by the tool. The score may be used by the provider with the ASA-PS assignment if they are in agreement with the recommendation.

We hypothesize that anesthesia and nonanesthesia providers at our institution underscore patients based solely on BMI. Additionally, we hypothesize that, unlike the findings from Fielding-Sing et al., the publication of examples by the ASA, as well as educational efforts, will have improved the accuracy and concordance of ASA-PS assignment over the eleven-year time period of this study. In 2015, our department started an initiative to improve the accuracy of ASA-PS assignment using a multipronged approach. We developed a finance committee with clinical, administrative, and revenue cycle representation that disseminated several emails in the fall of 2015 and spring of 2016 to department leaders highlighting the historical discordance in ASA-PS and its potential financial impact. We also had a department grand round dedicated to this topic in February 2016, placed laminated copies of the ASA definitions to assist with ASA-PS scoring in the spring of 2016, and developed a tool embedded within our EMR that assists the clinician with reviewing the patient's history and provides a recommended ASA-PS score based on identified comorbidities (Figures 1 and 2).

## 2. Methods

This retrospective observational study was deemed exempt from need to obtain informed consent by the Mayo Clinic Institutional Review Board. All patients over the age of 18 who consented to use of their clinical data and had surgical procedures under general anesthesia at Mayo Clinic in Rochester, MN, between January 1, 2010, and December 31, 2020, were retrospectively analyzed. Variables collected included age, height (cm), weight (kg), BMI, ASA-PS, and type of surgery performed.

### 2.1. Statistical Analysis

Patient characteristics are summarized using mean ± SD for continuous variables and frequency counts and percentages for categorical variables. A dichotomous variable was created to indicate patients whose ASA status was underscored based on obesity criteria. Patients with BMI ≥30.0 kg/m^2^ were considered underscored if their assigned ASA status was less than 2, and patients with BMI ≥40.0 kg/m^2^ were considered underscored if their assigned ASA status was less than 3. The percentage of patients underscored based on obesity criteria was calculated using different denominators (i.e., different patient populations) including all patients, all obese patients, and BMI subgroups of 30.0 to 39.9 kg/m^2^ and ≥40 kg/m^2^. The percentage of patients who were underscored is summarized overall and according to calendar year. We fit a segmented logistic regression model to estimate the trends (per-year change in odds) of ASA under classification according to the severity of obesity during 3 epochs: preimplementation (2010–2014), implementation (2015), and postimplementation (2016–2020) [25].

## 3. Results

Over our study period, 495,7779 patients were identified as having a procedure under general anesthesia at our institution with all available data present.  Table 1 shows patient demographics and breakdown of patients based on BMI. Demographic data show equal distribution of males (50%) to females (50%) in the general population studied.  Table 1 shows further breakdown of gender and its correlation to each level of obesity. The largest gender discrepancy was in the BMI >40.0 category where the population was 39% male and 61% female.

Of the patients included in the study, 160,844 (32.4%) met the criteria to be classified as a minimum ASA 2 based on BMI criteria (BMI 30.0–39.9 kg/m^2^), and 39,579 (8.0%) met the criteria to be classified as a minimum ASA 3 based on BMI criteria (BMI >40.0 kg/m^2^). Of the 160,844 patients with class 1 or 2 obesity (BMI 30.0–39.9 kg/m^2^), minimum ASA-PS 2, a total of 5,695 (3.5%) patients were underscored. In the 39,579 patients who met the criteria for class 3 obesity (BMI >40.0 kg/m^2^), minimum ASA-PS 3, there were 10,772 (27.2%) patients underscored. Within the class 3 obesity group, 388 (1.0%) of patients were underscored by 2 full categories as an ASA-PS I.

The preimplementation, implementation, and postimplementation per-year changes in odds of ASA under classification were 1.05 (1.03, 1.08), 0.72 (0.65, 0.79), and 0.94 (0.92 to 0.97) for patients with BMI 30 to 39.9 and 1.04 (1.02, 1.06), 0.53 (0.49, 0.58), and 0.87 (0.85, 0.89) for patients with BMI of 40 or more. Both the implementation and postimplementation per-year change in odds differed significantly compared to preimplementation for BMI groups (all *P* values <0.001).

When breaking down data year-to-year following the release of the updated ASA-PS in 2014, there was a statistically significant improvement in underscoring of patients based on BMI criteria alone (Table 2) from 2015 to 2020 in all obesity categories (*P* ≤ 0.001). Despite the improvement year-to-year in the population studied, patients undergoing general anesthesia during the study period of 2020 continued to be underscored. Those patients in 2020 who met criteria for class 3 obesity continued to be underscored at a higher rate (15.8%) than those with class 1 or 2 obesity (2.6%).

There was a statistically significant per-year increase in the odds of underscoring of ASA-PS based on BMI alone from 2010 to 2015 (*P* < 0.001); however, between-year variability was low (maximum rate minus minimum rate: 2.6%, 1.0%, and 1.5% among class 3, class 1-2, and all obese patients, respectively).

**Figure 3:**
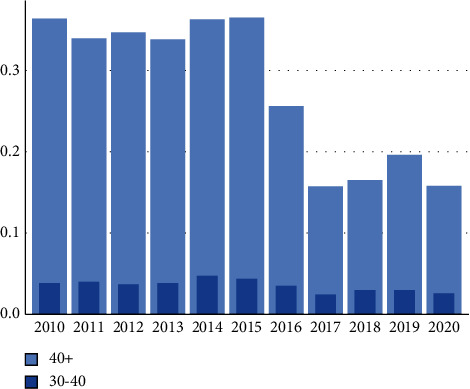
Bar graph showing underscoring of ASA-PS based on BMI criteria alone from 2010–2020 for BMI ranges of 30–40 kg/m^2^ and >40 kg/m^2^.

Improvement in scoring can be found after 2015 (Figure 3).

## 4. Discussion

This study shows that the ASA-PS continues to be underscored based on obesity, particularly for class 3 obesity (BMI >40.0 kg/m^2^). Additionally, there does seem to be a trend in improving the accuracy of ASA-PS scoring over time. The 2014 and 2020 ASA-PS updated guidelines specifically state minimum scores for patients with class 1 and 2 obesity (ASA II) and class 3 obesity (ASA III) [5]. These represent a floor or minimum ASA-PS, but the determined score may be higher depending on other patient characteristics or comorbidities. Thus, the problem of underscoring maybe even more substantial than this study indicates, as there could be patients with class 3 obesity that were scored as ASA-PS 3 based on other comorbidities but would have been scored lower if those comorbidities were not present.

Our study adds to a growing body of literature on the subject, as previous studies looking at the concordance of scoring using the provided examples have yielded mixed results. Several articles have suggested inconsistencies and discordance in assignments among both anesthesiologists and nonanesthesia providers [17, 19]. Hurwitz et al. [15] created a web-based questionnaire using 10 hypothetical cases in which both anesthesia-trained and nonanesthesia-trained clinicians were asked to assign an ASA-PS score in case scenarios using the ASA-PS classification system, and then rescoring a second time using ASA-PS approved examples and definitions. The authors demonstrated that providing publicly available ASA-approved examples significantly improved concordance of the assignment of the ASA physical status by both anesthesia-trained and nonanesthesia-trained providers. The number of correctly assigned cases increased from 5/10 to 7/10 cases after examples were provided for anesthesia-trained providers and 8/10 for nonanesthesia-trained providers. Not evaluated was a longitudinal evaluation of whether the examples improved concordance and accuracy.

Variability studies using kappa scores have further quantified the variation between providers with both theoretical [7, 9, 11, 16, 20] and actual patients [13, 14, 17, 22]. Much of the discordance is attributed to the subjective identification of patient comorbidities and the severity of each condition. However, there are several objective variables listed on the current ASA-PS Classification System Current Definitions and ASA-Approved Examples website. A few of the provided examples include objective data such as BMI thresholds, cardiovascular or cerebrovascular events within a defined time period, age of infants, or specific comorbidities such as uterine rupture, sepsis, dialysis, or COPD. It is important to note that the list of examples in the guidelines released in 2014 and 2020 is not comprehensive and not intended to supersede critical evaluation and designation by trained providers.

We are encouraged by the significant improvement in concordance and accuracy of ASA-PS scoring over time. In 2015, our institution undertook an educational initiative on this topic which included a grand round presentation, multiple conference presentations, a new Department finance committee, and work on an IT solution to provide a recommended PS and provided copies of the ASA-PS examples throughout our anesthetizing locations. Significant improvement in PS scoring based on BMI was seen from 2015–2017, but, without continued active educational activities, there has been no further improvement over the last several years. This represents an opportunity to reinstitute educational activity to encourage further improvements in accuracy and convergence of ASA scoring on the day of surgery.

The use of an automated software tool or ASA-PS scoring algorithm within an electronic record may be useful in lessening the number of patients who are underscored, based on fixed criteria, such as BMI. Such automated methods and systematic approaches to the use of the ASA-PS have been reported and are in development, including one within our institution. Further educational opportunities or IT solutions may be beneficial to help reduce the percentage of patients underscored using the ASA-PS based on BMI as well as other comorbidities.

## 5. Conclusion

Despite the updated ASA-PS released in 2014 and again in 2020 that included definitions and examples specific to ASA-PS and BMI [4, 5], our study shows there continues to be an underscoring of patients based on objective BMI data alone. Rates of underscoring in general and correlation to increased rate of underscoring in patients with BMI >40 kg/m^2^ are consistent with a large national study looking at the accuracy of providers scoring utilizing BMI [21]. The use of the ASA-PS to classify patients preoperatively has many potential implications and is used by anesthesia-trained and nonanesthesia-trained providers in a wide variety of settings in traditional ORs, procedural spaces, and additional environments utilizing both anesthesia and nonanesthesia providers. Additional education, tools, and awareness on the use of the updated ASA guidelines and examples, especially on more objective criteria such as those listed for BMI, may be warranted to help improve overall scoring accuracy. Future studies focusing on IT solutions and utilization of software to identify clinical data, patient characteristics, and ICD 10 codes may also allow for less subjectivity and improve accuracy and concordance in the assignment of scores.

## Figures and Tables

**Table 1:** Patient characteristics overall and according to body mass index category^*∗*^.

	Overall (*N* = 495,779)	Body mass index, kg/m^2^			
		<20 (*N* = 23,865)	20–29.9 (*N* = 271,491)	30–39.9 (*N* = 160,844)	40+ (*N* = 39,579)
Age, years	57 (17)	51 (20)	58 (18)	59 (15)	55 (14)
Gender^†^					
Female	245,602 (50%)	16,797 (70%)	131,062 (48%)	73,766 (46%)	23,977 (61%)
Male	250,166 (50%)	7,067 (30%)	140,422 (52%)	87,075 (54%)	15,602 (39%)
ASA physical status					
I	32,110 (6%)	2,191 (9%)	23,836 (9%)	5,695 (4%)	388 (1%)
II	215,120 (43%)	9,465 (40%)	124,951 (46%)	70,320 (44%)	10,384 (26%)
III	207,301 (42%)	9,862 (41%)	101,432 (37%)	71,529 (44%)	24,478 (62%)
IV	31,109 (6%)	1,778 (7%)	15,888 (6%)	9,980 (6%)	3,463 (9%)
V	10,139 (2%)	569 (2%)	5,384 (2%)	3,320 (2%)	866 (2%)

^*∗*^Age is summarized as mean (standard deviation). Categorical variables are summarized as number (percentage). ^†^Gender was missing for 11 patients.

**Table 2:** Number and percentage of patients with ASA status underscored based on obesity criteria in patients undergoing general anesthesia^*∗*^.

	All patients		All obese patients		Obese patients according to BMI			
					30.0 to 39.9 kg/m^2^		40.0 kg/m^2^ or more	
	*N*	# (%)	*N*	# (%)	*N*	# (%)	*N*	# (%)
Overall	495,779	16,467 (3.3%)	200,423	16,467 (8.2%)	160,844	5,695 (3.5%)	39,579	10,772 (27.2%)
Year								
2010	42,924	1,599 (3.7%)	16,502	1,599 (9.7%)	13,554	528 (3.9%)	2,948	1,071 (36.3%)
2011	43,591	1,639 (3.8%)	16,654	1,639 (9.8%)	13,453	554 (4.1%)	3,201	1,085 (33.9%)
2012	44,276	1,638 (3.7%)	17,160	1,638 (9.5%)	13,925	516 (3.7%)	3,235	1,122 (34.7%)
2013	45,673	1,785 (3.9%)	18,124	1,785 (9.8%)	14,560	579 (4.0%)	3,564	1,206 (33.8%)
2014	44,904	2,022 (4.5%)	17,989	2,022 (11.2%)	14,269	674 (4.7%)	3,720	1,348 (36.2%)
2015	43,880	1,993 (4.5%)	18,195	1,993 (11.0%)	14,473	637 (4.4%)	3,722	1,356 (36.4%)
2016	45,898	1,548 (3.4%)	19,266	1,548 (8.0%)	15,292	531 (3.5%)	3,974	1,017 (25.6%)
2017	47,416	1,035 (2.2%)	20,065	1,035 (5.2%)	15,904	385 (2.4%)	4,161	650 (15.6%)
2018	46,286	1,081 (2.3%)	19,040	1,081 (5.7%)	15,271	459 (3.0%)	3,769	622 (16.5%)
2019	48,469	1,212 (2.5%)	19,926	1,212 (6.1%)	16,120	467 (2.9%)	3,806	745 (19.6%)
2020	42,462	915 (2.2%)	17,502	915 (5.2%)	14,023	365 (2.6%)	3,479	550 (15.8%)

^*∗*^The number and percentage (# (%)) of patients underscored are presented overall, according to year of surgery. Data are presented for all patients, all obese patients, patients with a BMI of 30.0 to 39.9, and patients with a BMI of 40.0 or greater.

## Data Availability

The clinical data used to support the findings of this study are available from the corresponding author upon request.
